# Preoperative serum calcitonin may improve initial surgery for medullary thyroid cancer in patients with indeterminate cytology

**DOI:** 10.1111/ans.17690

**Published:** 2022-04-12

**Authors:** Karishma Jassal, Nandhini Ravintharan, Swetha Prabhakaran, Simon Grodski, Jonathan W. Serpell, James C. Lee

**Affiliations:** ^1^ Monash University Endocrine Surgery Unit Alfred Hospital Melbourne Victoria Australia; ^2^ Department of Surgery, Central Clinical School Monash University Melbourne Victoria Australia

**Keywords:** calcitonin, thyroid carcinoma, thyroidectomy

## Abstract

**Background:**

Medullary thyroid cancer (MTC) is rare, with poorer outcomes than differentiated thyroid cancer. We aimed to identify areas for improvement in the pre‐operative evaluation of patients with possible MTC in a high‐volume endocrine surgery unit in accordance with current practice guidelines. We hypothesised that the selective use of serum calcitonin (sCT) as a biomarker for possible MTC could guide the extent of initial surgical management.

**Methods:**

We recruited MTC patients between 2000 and 2020 from the Monash University Endocrine Surgery Unit database. Demographics, tumour characteristics, pre‐operative evaluation, operative management, and outcomes were analysed.

**Results:**

Of 1454 thyroid cancer patients, 43 (3%) had MTC. Pre‐operatively, 36 (84%) patients with MTC confirmed on cytology (28, 65%), elevated sCT (6, 14%) or *RET* mutation (2, 4%). Of these 36 patients, 31 (86%) had optimal extent of thyroidectomy and lymph node dissection (LND). Five (14%) had less than total thyroidectomy due to nerve injury. Thirty‐four patients had compartmental LND. In the 12 (27%) patients with indeterminate or non‐diagnostic cytology, 5 had elevated sCT and were managed as above. None of the remaining seven had LND, thus potentially suboptimal surgery.

**Conclusion:**

Our findings reflect the rarity of MTC, and the challenges of pre‐operative diagnosis. The addition of sCT may improve surgical planning in patients with indeterminate cytology.

## Graphical abstract

Medullary thyroid cancer is a challenging and rare diagnosis. There are conflicting recommendations as to the use of sCT between different guidelines and so we hoped to identify the utility of serum calcitonin as a biomarker for possible MTC.

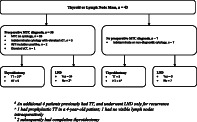

## Introduction

Medullary thyroid cancer (MTC) is a rare diagnosis, which accounts for 2–3% of all thyroid malignancies.^1^, ^2^ The diagnosis and management of MTC can be challenging. MTC arises from thyroid parafollicular calcitonin secreting cells, with increasing levels of serum calcitonin (sCT) serving as a biomarker for tumour burden. Hereditary MTC is described in 14–52% of patients and sporadic MTCs in 48–86% of patients.^3^ In both forms, regional nodal metastases have been reported to occur in up to 80% of patients and approximately 20% have distant metastases, at presentation. The average 5‐year survival rate for MTC is 73–97% and predictors of survival include TNM stage, disease extent at diagnosis as well as extent of thyroidectomy.^4^, ^5^, ^6^, ^7^ Therefore, accurate pre‐operative diagnosis is crucial in guiding expedient and precise initial management, which would confer improved disease‐free and overall survival. A pre‐operative diagnosis can often be established by fine needle aspirate (FNA) cytology, sensitivity of which rangers from 50% to 80%. Though, higher sensitivity can be attained by the addition of calcitonin measures.^8^

At present, the American Thyroid Association (ATA) 2015 guidelines does not recommend either for or against routine measurement of sCT in patients with thyroid nodules. This is contrary to that of the European Thyroid Association (ETA) consensus, which highlight sCT as an important diagnostic tool and recommends its measurement in the initial workup of all thyroid nodules.^9^, ^10^, ^11^ In this study, we aimed to determine the role of sCT in the workup of patients with thyroid nodules, in the context of these practice guidelines. We hypothesised that a selective approach to measuring sCT may improve the pre‐operative diagnosis of MTC and initial surgical planning.

## Methods

This was a retrospective study of all patients who underwent surgery for MTC within the Monash University Endocrine Surgery Unit (MUESU) from January 2000 to June 2020, conducted under institutional review board approval (HREC 784/19). Patients with MTC on histopathology reports or known *RET* mutation were recruited from the prospectively maintained institutional database. Patients were included if they were investigated for a thyroid nodule or neck mass for the first time at our institution. Data on patient demographics, pre‐operative cytology, sCT, tumour characteristics, operative management and outcomes were collected and analysed. Pre‐operative FNA cytology, when performed, was reported according to the Bethesda system, or in a similar structure.^12^ Pre‐operative sCT measurements were obtained at the treating clinician's discretion. Operative specimens were subjected to standard histopathological analysis as well as immunohistochemistry stains with calcitonin, chromogranin, synaptophysin, TTF‐1, pancytokeratin and thyroglobulin. Disease‐free survival (DFS) was defined as the absence of clinical or radiological structural recurrent disease.

Descriptive data are presented as mean ± standard deviation (SD) if the data are normally distributed, or median and interquartile range (IQR) as appropriate. Survival curves were plotted using the Kaplan–Meier method. All statistics were performed using Prism version 9.0.0, GraphPad Software.

## Results

A total of 1454 procedures for thyroid malignancies were identified and MTC accounted for 43 (3%) of those cases.

### Demographics

The mean age of the 43 MTC patients was 57.3 ± 17.6 years at the time of diagnosis (Table 1). Gender distribution was relatively even with a slight predilection for females (56%). Seven (16%) patients had a family history of thyroid malignancy—four with family history of MTC and *RET* mutation and three with family history of non‐MTC thyroid malignancy. Eighteen patients had *RET* germline mutation testing due to either young age at presentation or suspicious clinical features of multiple endocrine neoplasm—two patients pre‐operatively and 16 patients post‐operatively—of these, four (9%) patients were positive for a *RET* germline mutation.

**Table 1:** Clinical details and tumour staging (*n* = 43)

Clinical details		Pre‐op Dx (*N* = 36)	Post‐op Dx (*N* = 7)	*P*
Mean age in years (SD)	57.3 (17.6)	58.5 (17.6)	46 (24.7)	0.4
Female, n (%)	24 (55.8)	21 (87.5)	3 (12.5)	0.7
Sporadic cases, n (%)	36 (83.7)	29 (80.5)	7 (19.4)	
*RET* germline mutation positive, n (%)	4 (9.3)	4	‐	
Family history of non‐medullary thyroid malignancy, n (%)	3 (7.0)	1 (33.3)	2 (66.7)	
Median maximal tumour size in mm (IQR)	25 (15–47)	35 (13–60)	20 (17–35)	0.3
Tumour stage, *n* (%)				
Tx*	6 (14.0)	6 (14.0)	–	
T1a	4 (9.3)	3 (75)	1 (25)	
T1b	10 (23.3)	7 (70)	3 (30)	
T2	9 (20.9)	6 (67)	3 (33)	
T3a	10 (23.3)	10	–	
T3b	1 (2.3)	1	–	
T4a	2 (4.7)	2	–	
T4b	0	–	–	
Nodal stage, *n* (%)				
N0	19 (44.2)	12 (63)	7 (37)	
N1a	6 (14.0)	6	–	
N1b	17 (39.5)	17	–	
Metastasis, *n* (%)				
M0	38 (88.4)	31 (82)	7 (8)	
M1	4 (9.3)	4	–	
TM Stage, *n* (%)				
I	9 (20.9)	5 (56)	4 (44)	0.01
II	10 (23.3)	7 (70)	3 (30)	
III	6 (14.0)	6	–	
IVa	15 (34.9)	15	–	
IVb	1 (2.3)	1	–	
IVc	1 (2.3)	1	–	
Could not be staged*	1 (2.3)	1	–	

Abbreviations: SD, standard deviation; IQR, interquartile range.

* Unable to determine due to missing data.

### Pre‐operative evaluation

In this study, 36 of 43 patients (84%) had a pre‐operative diagnosis of MTC—28 on cytology, 6 on elevated sCT and 2 on *RET* mutations.

The majority of patients (40, 93%) had an FNA prior to surgery. Of the remaining three patients, 2 had known positive *RET* mutation preoperatively and 1 had elevated sCT in the context of previous MTC diagnosis. Of the 40 patients with FNA, 28 (70%) had cytology consistent with MTC, and 12 (30%) had indeterminate or non‐diagnostic cytology (Bethesda 1 = 2; Bethesda 3 = 1; Bethesda 4 = 3; Bethesda 5 = 6).

Overall, 30 (70%) patients had sCT measured pre‐operatively. Five patients out of the 12 with indeterminate cytology, and another with a neck mass subsequent to previous thyroidectomy for MTC, had sCT levels pre‐operatively. They all had elevated sCT, ranging from 26 to 8800 pmol/L (reference range < 4.0 pmol/L).

### Surgical treatment and histology

Thirty patients (70%) had a total thyroidectomy (TT), 9 patients (21%) had a hemithyroidectomy (HT) and 4 patients (9%) had a lymph node dissection (LND) alone for recurrence (all had TT at another institution previously). Of the nine patients who had HT, five had pre‐operative diagnosis of MTC therefore also had central LND. However, contralateral thyroidectomy was abandoned due to recurrent laryngeal nerve (RLN) injury indicated by intraoperative neuromonitoring (IONM) (four cases) or nerve sacrifice (one case). None of them proceeded to completion thyroidectomy at a later date due to advanced metastatic disease,^1^ patient decision^3^ and death from lymphoma.^1^

A total of 34 patients (79%) underwent LND. Of these, 19 patients (56%) underwent central LND only, and 15 patients (44%) underwent both central and lateral LND (*n* = 11), or lateral LND alone for recurrence (*n* = 4). Eighteen (53%) patients had positive lymph nodes.

Of the six patients with an elevated pre‐operative sCT, three had a TT and LND; one had LND for recurrence; one had TT without LND due to absence of visible lymph nodes to dissect; the remaining patient was planned for a TT with central LND, but a RLN palsy recognized by IONM resulted in a HT with central LND instead. As the disease burden was low, a decision was made for ongoing surveillance rather than completion thyroidectomy according to the patient's preference.

**Fig. 1:**
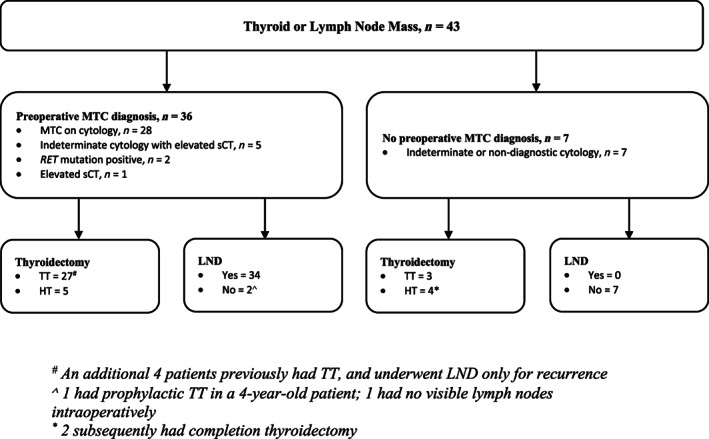
Preoperative evaluation of thyroid nodule and initial surgical management. *TT*, total thyroidectomy; *HT*, hemithyroidectomy; *LND*, lymph node dissection. ^#^ An additional four patients previously had TT, and underwent LND only for recurrence. ^∧^ one had prophylactic TT in a 4‐year‐old patient; one had no visible lymph nodes intraoperatively. * two subsequently had completion thyroidectomy.

Of the seven patients with indeterminate cytology who did not have pre‐operative sCT, three underwent TT—two for goitres and one for Bethesda 5 cytology (suspicious for malignancy). The other four underwent diagnostic HT—two of whom subsequently underwent completion surgery. None of them had a LND in their index operation (Fig. 1).

The median maximal dimension of the primary tumour was 25 (IQR 15–47) mm. Just over half of the patients (53%) had advanced disease (Stage III or IV) at the time of presentation (Table 1). The maximal median dimension of the primary tumour was 35 (IQR 13–60) mm in the 36 patients with pre‐operative MTC diagnosis, and 20 (IQR 17–35) mm in the 7 without pre‐operative diagnosis (*P* = 0.3). All the patients without pre‐operative diagnosis had either Stage I or II disease, and the distribution of disease stage were different between those with and without pre‐operative diagnosis (*P* = 0.01).

### Complications and outcomes

Temporary recurrent laryngeal nerve palsy (RLNP) occurred in 6 (14%) and permanent in 3 (7%) patients. All permanent RLNP were anticipated as the nerves were intentionally sacrificed due to heavy tumour burden. Three (7%) patients developed temporary hypocalcaemia, and permanent hypoparathyroidism occurred in 1 (2%) patient. Two (4%) patients required return to theatre—one (2%) had a neck haematoma requiring evacuation, and the other (2%) developed a chyle leak from a minor lymphatic branch. Eight (18%) patients received adjuvant therapy post‐operatively—5 (11%) had external beam radiotherapy (EBRT) and 3 (7%) had both a tyrosine kinase inhibitor and EBRT.

**Fig. 2:**
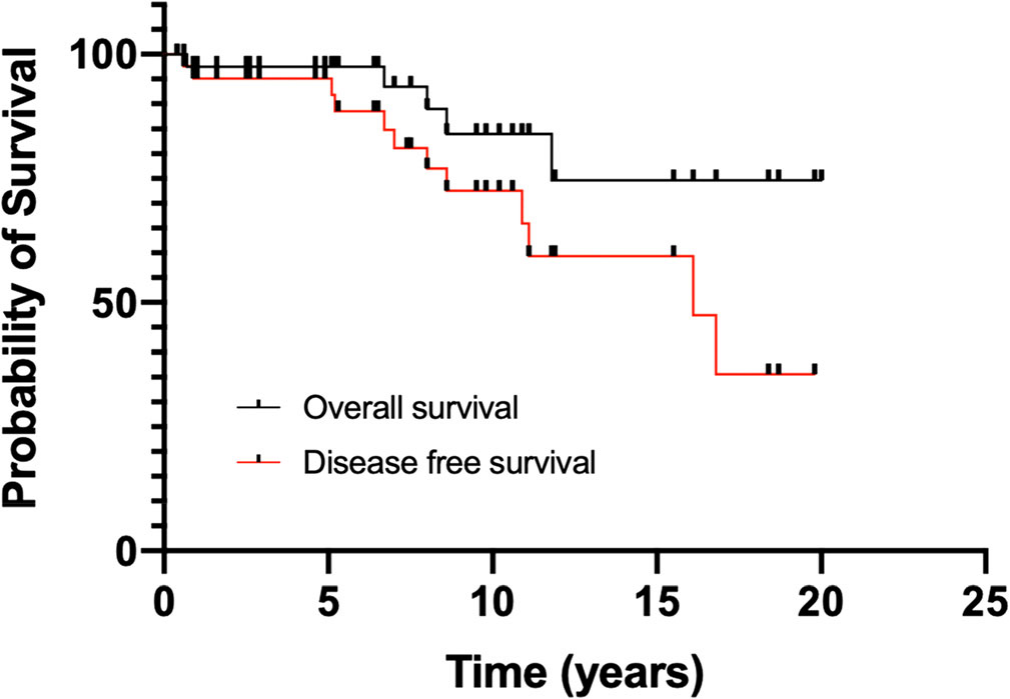
Overall survival and disease‐free survival (*n* = 43).

Median follow‐up for the study cohort was 7.7 (IQR 3.9–11) years. At last follow‐up, 22 (51%) patients were alive without disease, 6 (14%) were alive with evidence of disease, 7 (16%) patients had died of MTC, and 8 (18%) patients were lost to follow‐up or died of other diseases. Overall survival (OS) was 97% at 5 years, while disease‐free survival (DFS) was 91% at 5 years (Fig. 2). Both OS and DFS declined significantly in the interval between 10 and 15 years, although less than half of the patients within this cohort were followed this expansively (*n* = 14).

## Discussion

In this retrospective study of 43 patients with confirmed MTC on histopathology or *RET* mutation, 28 (70%) had a pre‐operative diagnosis of MTC on cytology, and 2 based on *RET* mutation. A further 6 had their diagnosis confirmed by elevated serum calcitonin. All 36 patients with pre‐operative diagnosis of MTC were recommended the optimal, guideline‐driven operation. All 36 had the appropriate lymph dissection, but 5 had hemithyroidectomy due to nerve injury—resulting in 31 (86%) achieving that optimal initial surgery. Of the seven patients without preoperative diagnosis of MTC, none received the optimal initial surgery—none had a lymph node dissection and four had a diagnostic hemithyroidectomy.

An accurate pre‐operative diagnosis and appropriate initial surgery are crucial components of the treatment for MTC. It is well accepted that the minimum extent of surgery for MTC is TT with central LND.^9^, ^13^, ^14^, ^15^ The present study was undertaken to determine the impact of pre‐operative diagnosis of thyroid nodules on the adequacy of the index operation for patients with MTC. All of the patients with a pre‐operative diagnosis of MTC were recommended total thyroidectomy and the appropriate nodal dissection during index operation as per international guidelines.^9^, ^10^, ^15^ However, in the absence of a pre‐operative diagnosis none of them had the optimal index operation.

Retrospective studies have suggested that patients who underwent initial TT with central LND required fewer subsequent operative procedures compared to patients who originally had lesser procedures.^16^, ^17^, ^18^ Further, it is established that sCT levels are unlikely to normalize in patients who require reoperation for either persistent or recurrent disease despite multiple reoperations.^19^ This emphasizes that achieving the optimal index operation confers the best chance of cure. It should be noted that for patients whose MTC diagnosis was made after HT, completion thyroidectomy is recommended if there is a *RET* mutation, elevated sCT level, or residual disease on imaging.^9^ This was the case in two of the four patients in this study who had a HT without LND. Based on these recommendations, it can be intimated that achieving the appropriate compartmental LND in the index operation, especially of the central nodes, is a crucial step.

It can be difficult to determine retrospectively how the decision‐making process might have been changed if additional information was available—in this case, sCT levels. Of the 36 patients with preoperative MTC diagnosis, 31 (86%) had the optimal surgery with total thyroidectomy and appropriate compartmental lymph node dissection. There is no doubt that the only reason preventing the other five from having the optimal initial surgery was RLN injury. The morbidity of a potential bilateral RLN palsy was considered too great a risk in these patients who had hemithyroidectomy, given the known disease burden. On the other hand, none of the seven patients without preoperative diagnosis of MTC had lymph node dissection. It is possible that they had all had suboptimal surgery. From the findings of this study, it can also be said that it is very likely that six patients were recommended optimal surgery purely based on the elevated sCT levels.

Routine use of sCT as part of thyroid nodule workup has been a topic of controversy for many years. While it is recognized that the early diagnosis with sCT may confer improved cure rates in patients with PTC, the American Thyroid Association does not recommend its routine use in their 2015 revised MTC guidelines. This is due to concerns of cost‐effectiveness, and the uncertainty of the clinical significance and natural history of MTC diagnosed by sCT.^9^, ^10^ On the contrary, sCT is recommended as a part of the initial investigations of thyroid nodules in the European consensus statement for management of differentiated thyroid carcinoma.^11^ Interestingly, there are studies from America that suggest routine sCT in the workup of thyroid nodules is in fact cost‐effective; to a level comparable to colonoscopy and breast screening programs.^20^, ^21^ Early diagnosis and avoidance of two‐stage thyroidectomy were cited as the main reasons for the cost‐effectiveness.^20^ However, it is suggested that its adoption and cost‐effectiveness should be assessed with local prevalence and cytology detection rate in mind.^21^ In addition, cost‐effectiveness aside, as clinicians, we need to consider the moral and ethical aspects of patient care in the context of local resources.

Thyroid FNA cytology is indeterminate or non‐diagnostic in up to 25% of clinically significant nodules. In a previous study from our institution, the rate of non‐diagnostic or indeterminate cytology (Bethesda categories 1, 3, 4 and 5) was 32%. This higher percentage reflects the surgical cohort as many patients with benign cytology were manage non‐operatively.^20^ In the same study, Stewart *et al* revealed that patients with DTC are significantly more likely to receive the optimal extent of initial surgery if a definitive pre‐operative diagnosis is reached.^22^ This highlights the importance of using adjuncts such as sCT and/or carcinoembryonic antigen to clinch a diagnosis of MTC when cytology is indeterminate. The four patients with recurrence included in this study also demonstrate the importance of confirming the diagnosis of MTC recurrence either on cytology (*N* = 3) or elevated sCT in conjunction with anatomical imaging (*N* = 1) before proceeding with lymph node dissection. Since these patients did not undergo surgery for their primary disease at our institution, it was important to investigate their recurrent neck mass systematically.

Our cohort of MTC patients reflects the current literature; they accounted for 3% of our institute's thyroid malignancies, consistent with the 3–10% quoted in other epidemiological studies.^2^, ^23^, ^24^ The mean age of diagnosis in this study's patient cohort is slightly older to that in other studies which typically reported patients in their fourth to fifth decade.^9^, ^25^, ^26^ This is likely because most of the patients in our cohort had sporadic forms of MTC which is known to present later in life as compared to inherited MTC.^27^

Of the 40 patients who had pre‐operative FNA, the 65% who had a cytological diagnosis of MTC is somewhat higher compared to other studies. In a large multicentre study by Essig *et al*. with 313 MTC patients, involving 12 institutions over 29 years, the cytological diagnostic rate of MTC was 46% in the 245 patients who had an FNA.^28^ A meta‐analysis of 15 papers reported that only 56% of histologically proven MTCs are correctly detected by cytologic evaluation or reported as possible MTC.^29^ The higher proportion of patients with a pre‐operative FNA diagnosis in our cohort might reflect a referral bias for patients with known MTC presenting to our referral centres.

We also noted in this study that nearly half (44%) of the patients presented with disease limited to the thyroid without any lymph node involvement (Stage I and II disease). This figure is similar in comparison to the other studies, where reported rates of localized disease range from 39% to 49%.^17^, ^30^, ^31^ Despite the high proportion of Stage I and II disease, higher RLNP rates were seen in MTC patients, compared to our overall palsy rates.^32^ Although studies have shown that adding a central neck LND did not increase the risk of RLNP in general,^33^, ^34^ surgeons likely adopt a more aggressive surgical approach when treating patients with known MTC—both in skeletonising or sacrificing the RLN during dissection. This might have contributed to the higher palsy rate.

It is worth noting that the 94% 5‐year OS is comparable to the 97% reported in the UK^35^; both of which are somewhat higher than the 89% reported using the National Cancer Institute Surveillance, Epidemiology, and End Results (SEER) data. The significance of this observation and the underlying reasons both need further investigation. However, an American study by Al‐Qurayshi *et al*. demonstrated that patients who were without health insurance had a lower survival rate compared to patients with private insurance even after controlling for age, comorbidities and stage.^36^ Similar associations between outcomes and insurance status have been reported for all thyroid cancers in general in the USA.^37^ It is possible that universal healthcare systems, such as the Australian Medicare scheme and the NHS in the UK, contribute to this observation.

There were some limitations in this study. Due to its retrospective nature, it is susceptible to limitations of a retrospective study such as incomplete medical records. There is the possibility of selection bias by clinicians regarding pre‐operative assessment, surgical and adjuvant therapy. Additionally, not all patients in our cohort had their germline *RET* mutation status tested. Despite the long period included in this study, the follow up period varied significantly, and the sample size was small due to the rarity of MTC. For these reasons, we were not able to stratify survival patterns according to preoperative or postoperative sCT, stage of disease, or *RET* mutation status. Due to the small sample size of this study, the results and conclusions should be interpreted with this in mind. Future multicentred studies with clinical outcomes and cost–benefit analysis may be able to provide more clarity in the Australasian setting.

## Conclusion

Our findings reflect the rare entity that is MTC and its challenges in diagnosis. From an observational standpoint, sCT improves pre‐operative evaluation of MTC presenting as indeterminate cytology and aids operative planning. While we cannot form strong conclusions or recommendations based on this study alone, we believe that there is an argument for more liberal use of sCT in the workup of cytologically indeterminate thyroid nodules in select patients, particularly when there is clinical, radiological or cytological suspicion of malignancy. Although the selection criteria in addition to indeterminate cytology, as well as cost‐effectiveness in the Australian setting require further investigation, we believe this practice may lead to better tailoring of the index surgery, which may further lead to reduced morbidity and even mortality.

## Conflict of interest

None declared.

## Author contributions

**Karishma Jassal:** Conceptualization; data curation; formal analysis; writing – original draft; writing – review and editing. **Swetha Prabhakaran:** Formal analysis; investigation; resources. **Simon Grodski:** Conceptualization; methodology; supervision; writing – review and editing. **Jonathan W. Serpell:** Conceptualization; methodology; supervision; writing – review and editing. **Nandhini Ravintharan:** Data curation; investigation, project administration, writing – original draft, writing – review and editing. **James Lee:** Conceptualization, funding acquisition, methodology, project administration, supervision, writing – review and editing.
